# The Inhibition Effect of Tert-Butyl Alcohol on the TiO_2_ Nano Assays Photoelectrocatalytic Degradation of Different Organics and Its Mechanism

**DOI:** 10.1007/s40820-015-0080-2

**Published:** 2016-01-12

**Authors:** Xuejin Li, Jinhua Li, Jing Bai, Yifan Dong, Linsen Li, Baoxue Zhou

**Affiliations:** 1 School of Environmental Science and Engineering, Shanghai Jiao Tong University, No. 800 Dongchuan Rd, Shanghai, 200240 People’s Republic of China; 2 Key Laboratory for Thin Film and Microfabrication of the Ministry of Education, Shanghai, 200240 People’s Republic of China

**Keywords:** Tert-butyl alcohol, Photoelectrocatalysis, TiO_2_ nano assays, Hydroxyl radical inhibitor, Inhibition effect

## Abstract

The inhibition effect of tert-butyl alcohol (TBA), identified as the ^•^OH radical inhibitor, on the TiO_2_ nano assays (TNA) photoelectrocatalytic oxidation of different organics such as glucose and phthalate was reported. The adsorption performance of these organics on the TNA photoelectrode was investigated by using the instantaneous photocurrent value, and the degradation property was examined by using the exhausted reaction. The results showed that glucose exhibited the poor adsorption and easy degradation performance, phthalate showed the strong adsorption and hard-degradation, but TBA showed the weak adsorption and was the most difficult to be degraded. The degradation of both glucose and phthalate could be inhibited evidently by TBA. But the effect on glucose was more obvious. The different inhibition effects of TBA on different organics could be attributed to the differences in the adsorption and the degradation property. For instance, phthalate of the strong adsorption property could avoid from the capture of ^•^OH radicals by TBA in TNA photoelectrocatalytic process.

## Introduction

Titanium dioxide (TiO_2_) has been demonstrated to be a promising and cost-effective alternative material in the photoelectrocatalytic (PEC) treatment of wastewater that containing refractory pollutants since it was observed [1–8]. As a typical photoanode material, the TiO_2_ nano assays (TNA) process the advantages such as the uniform distribution, neat arrangement, large specific surface area, and strong adsorption ability. Therefore, the TNA electrode shows relatively more excellent PEC performance and conversion efficiency comparing with other nano-TiO_2_ film materials. For this reason, the TNA preparation and its application to pollutants degradation have drawn lots of concerns [9–13].

The PEC performance could be affected by kinds of factors. As known, the type of photocatalyst is a crucial factor in PEC degradation of organics, and lots of efforts have been made to improve the photocatalyst including the structure, the modification on the surface, and so on [5]. Furthermore, the configuration of reactor is an important factor in the PEC performance, and many researches have been carried on to improve the efficiency of the reactor [14]. In addition, the reactants composition of the reaction system could also affect the PEC performance. It has been found that some small molecule organics could increase the degradation rate of other organics. For example, methanoic acid could enhance the photocurrent values and the reaction activity in the PEC process [15]. However, other chemical substances such as tert-butyl alcohol (TBA), phosphate, and carbonate could inhibit the hydroxyl radical activity of ozone oxidation process [16].

The effects of TBA on the ozone and Fenton oxidation processes for organics degradation have been studied in detail relatively [17–19]. Staehelln and Holgne found that TBA served as the ^•^OH radical inhibitor and inhibited the transition path from O_2_ to peroxy radical in the O_3_ decompose process [16]. It has been reported that TBA separated the direct molecular ozone reaction pathway in humic acid oxidation by O_3_ [20]. Dao and Laat found that TBA seriously inhibited the reaction of the hydroxy radicals in the degradation processes of atrazine, fenuron, and parachlorobenzoic acid by FeIINTA/O_2_, FeIINTA/H_2_O_2_, and FeIIINTA/H_2_O_2_ Fenton reaction [21]. However, the inhibition effect and mechanism of TBA in the PEC degradation process, especially on the surface of TNA, which is an important advanced oxidation method, has little or no description.

In the present work, the inhibition effects of TBA on different organics, including the weak adsorption of glucose and strong adsorption of phthalate, were studied, and the mechanism on the surface of the TNA in the PEC oxidation was reported. The adsorption and degradation performance of different organics on the surface of TNA were also investigated.

## Experimental

### Material and Sample Preparation

Unless otherwise indicated, all the reagents were analytical reagent grade and purchased from Sinopharm Chemical Reagent Company (Shanghai, China). Potassium hydrogen phthalate was used as the representative of phthalate. All solutions were made up with high-purity deionized water (18 MΩ) purified from a Milli-Q purification system (Millipore Corporation, Billerica, MA), and a NaNO_3_ solution served as the supporting electrolyte in samples.

### Preparation of the TNA Electrode

**Fig. 1:**
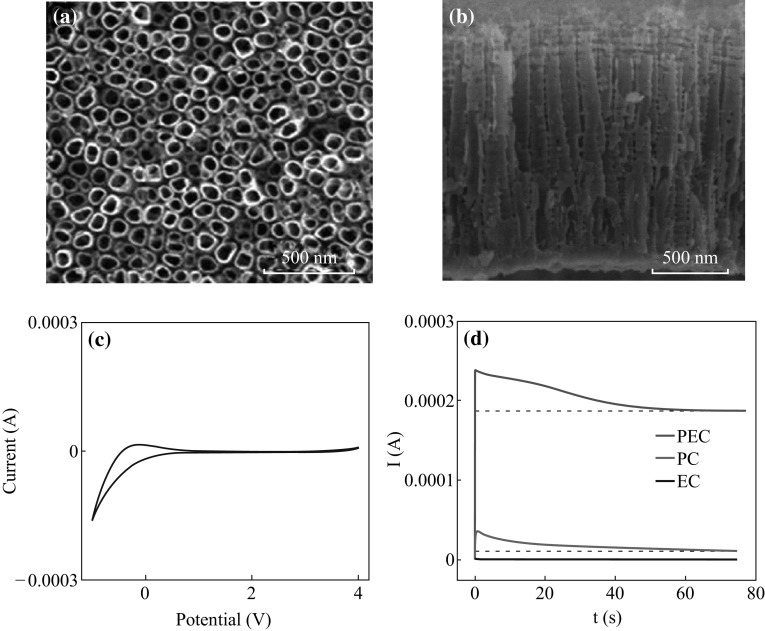
SEM images of the TiO_2_ nanotube arrays obtained from **a** the *top view* and **b** the cross-section. **c** The cyclic voltammetry performance of this thin-layer reactor. **d** The comparison of PEC, photocatalytic (PC), and electrocatalytic (EC) degradation of 100 mg L^−1^ glucose

The TiO_2_ nanotube arrays electrodes used in this work were prepared by the electrochemical anodic oxidation method [22]. The anode and cathode were titanium and platinum, respectively. The titanium was put into a mixture of 1 mol L^−1^ NaF, 1 mol L^−1^ NaHSO_4_, and 0.2 mol L^−1^ trisodium citrate, and NaOH was added to adjust the pH. The titania nanotube electrodes were prepared under constant stirring for 6 h with an applied bias of 20 V. They were then annealed in a laboratory muffle furnace at 500 °C for 3 h to form TNA. The SEM images of top view and the cross-section of the obtained TNA are shown in Fig. 1a, b, respectively. It can be seen that the TiO_2_ nanotubes are highly ordered and well aligned. The cyclic voltammetry performance of the thin-layer reactor used in this work is shown in Fig. 1c. It can be seen that the potential value of 2 V is in the range of redox potential window. At the same time, this potential value of 2 V could ensure the transfer of generated electrons to the external circuit. Considering above mentioned, the applied potential value of 2 V has been chosen in this work. Figure 1d shows the *I*–*t* curves obtained from the photocatalytic (PC) and electrocatalytic (EC) degradation of 100 mg L^−1^ glucose. Both the performance of the PC and EC degradation are ineffective on the degradation of organic, such as the much smaller current values than the PEC degradation. It illustrates that the potential and UV light illumination play a synergistic effect in PEC degradation of glucose (and also other organics matters).

### Reactor Used in the Experiment

**Fig. 2:**
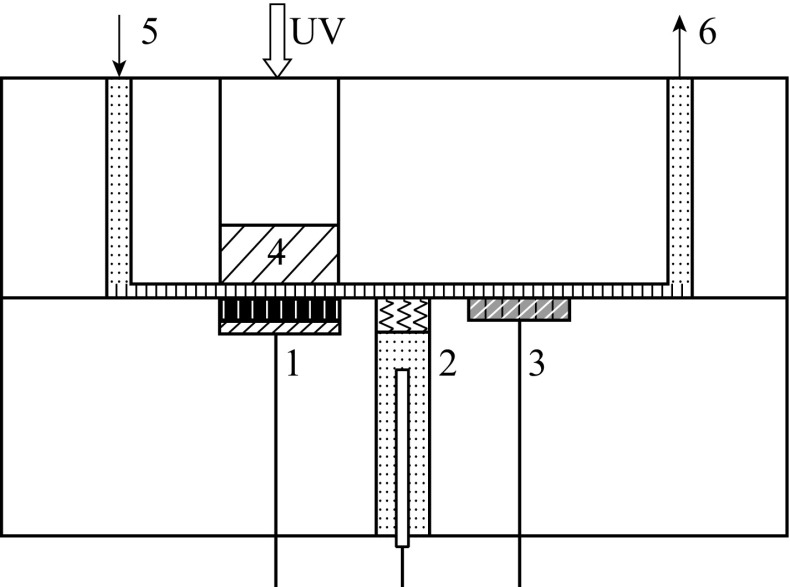
The structure of the thin-layer reactor. *1*—TiO_2_ nanotube array electrode; *2*—The saturated Ag/AgCl reference electrode; *3*—The Pt counter electrode; *4*—The quartz window; *5*—The flow inlet; *6*—The flow outlet

The photoelectron chemical experiment was carried out in a thin-layer reactor as shown in Fig. 2 [23]. As can be seen, the thin-layer reactor was a three-electrode system containing six main sections: the TNA anode electrode, the saturated Ag/AgCl reference electrode, the Pt counter electrode, the flow inlet and outlet, and a quartz window with a diameter of 1 cm. Two polytetrafluoroethylene planks were combined together to form a reaction cell. The thickness of the cell was controlled as only 0.1 mm to shorten the time and distance of the mass transfer from the bulk solution to the surface of the TNA electrode; meanwhile, it could also ensure the light transmittance of a 365 nm ultraviolet light-emitting diode (LED). The potential and current of the working electrode were controlled by an electrochemical workstation (CHI 610D, Shanghai) which linked with the computer to record the photocurrent response signals.

### Concentration Unit and Degradation Efficiency

The concentration unit of organics in oxygen equivalent (mg L^−1^) was used, and the computation method of the degradation efficiency was established based on the principle of PEC oxidation of organics in the thin-layer reactor.

The oxidation of organics in the photocatalytic process could be described as [24]:

1 $$ \begin{aligned} {\text{C}}_{y} {\text{H}}_{m} {\text{O}}_{j} N_{k} X_{q} + \left( { 2y - j} \right){\text{ H}}_{ 2} {\text{O }} \to y{\text{CO}}_{ 2} + qX^{ - } + k{\text{NH}}_{ 3} + \, \left( { 4y - 2j + m - 3k} \right){\text{ H}}^{ + } \hfill \\ + \, \left( { 4y - 2j + m - 3k - q} \right)e^{ - } \hfill \\ \end{aligned} $$

where *N* and *X* represent a nitrogen and a halogen atom, respectively, and *y*, *m*, *j*, *k*, and *q* represent the numbers of carbon, hydrogen, oxygen, nitrogen, and halogen atoms, respectively.

The theoretical value of the charge quantity generated in the organics PEC oxidation process could be defined as *Q*_th_

2 $$ Q_{\text{th}} = n{FVC}, $$

where *n* (*n* = 4*y*−2*j* + *m*−3 *k*−*q*) represents the amount of transferred charge generated by the unit mole organic oxidation, *F* is the Faraday constant, and *V* and *C* are the sample volume and the molar concentration, respectively.

The *Q*_th_ could be calculated according to Eq. (2) when the organics are completely degraded (namely exhausted oxidation) under the condition that the volume of the reactor is fixed and the species and the contents are known. Accordingly, the stoichiometric concentration of the organics could be represented by the captured charges in the PEC oxidation process. Take glucose as an example:

3 $$ {\text{C}}_{ 6} {\text{H}}_{ 1 2} {\text{O}}_{ 6} + {\text{ 6H}}_{ 2} {\text{O }} \to {\text{ 6CO}}_{ 2} + {\text{ 24H}}^{ + } + {\text{ 24e}}^{ - } $$

This equation suggests the mineralization of 1 mol of glucose generates 24 mol electrons. Thus, the Faraday’s law can be written as *Q*_th_ = 24*FVC*. Accordingly, the amount of glucose can be represented by the amount of the net charges. The conversion relationship 4e^−^ + 4H^+^ + O_2_ → 2H_2_O indicates that 4 mol of electrons is equivalent to 1 mol of O_2_. Therefore, the quantity of net charges could be represented by the oxygen equivalent (mg L^−1^) which has been used as the measurement unit of the organics concentration for the purpose of comparison in this work.

**Fig. 3:**
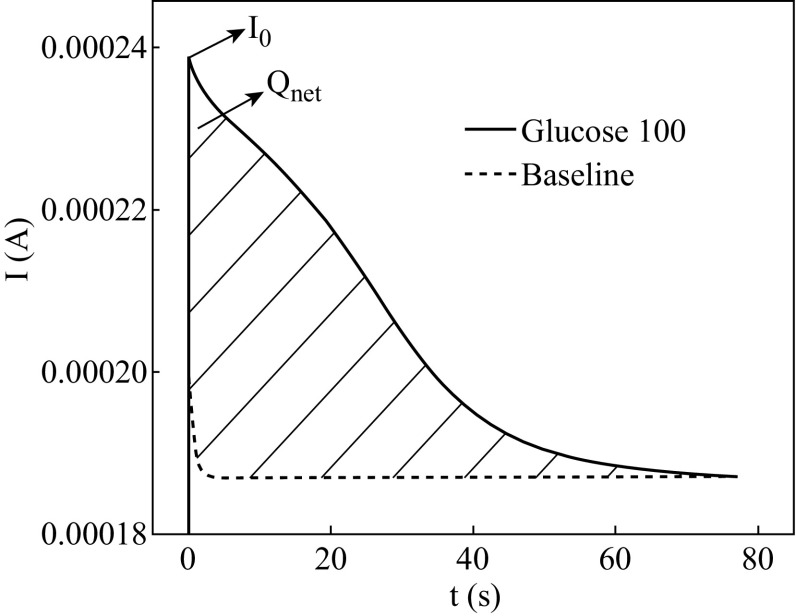
The schematic diagram of typical response photocurrent signals in the glucose oxidation

In the PEC process, the net charge (*Q*_net_) of organics could be calculated from the shaded area of the response signals, as is shown in Fig. 3. The baseline represents the response signals of the electrolyte containing none organics, and the shaded area between the peak and the baseline represents the charge generated by the organics sample, which also contains electrolyte.

4 $$ Q_{\text{net}} = \, \smallint Idt. $$

When the organics are incompletely degraded in the thin-layer reactor, the degree of degradation (*α*) could be obtained from the ratio of the captured *Q*_net_ and the theoretical value of the net charge quantity (*Q*_th_) of the sample:

5 $$ \alpha = Q_{\text{net}} /Q_{\text{th}} . $$

Based on the measurement of the total number of photoelectrons generated from the photocatalytic oxidation of organics, the photoelectrochemical method can easily quantify the degree of the oxidation according to Faraday’s law, assuming that we can ignore the complicated interim reactions in the traditional photoelectrochemical oxidation method. The rate of electron capturing (i.e., the value of the photocurrent) can directly describe the photocatalytic degradation efficiency.

It has been reported that complete PEC degradation of the glucose in the thin-layer reactor could be achieved [25]. Therefore, the quantity of transferred charges (*Q*_net-glucose_) in the glucose oxidation process could be recognized as *Q*_th_ to measure the oxidation extent of organics in this work. Figure 3 shows the schematic diagram of photocurrent signals of the glucose oxidation. As can be seen, the degradation situation is reflected by the *I*–*t* curve which is smooth and not fluctuating.

## Results and Discussion

The PEC degradation characteristics of organics on the surface of catalyst could be revealed easily by using a thin-layer reactor since both the mass transfer distance and the time it takes to travel from the solution to the surface of the electrode should be shorten. Thus, the oxidation of organic can be carried out quickly in the thin-layer reactor, which is conducive to examine the adsorption and degradation processes of organics on the surface of the catalyst [26]. On the contrary, in a bulky reactor, the large reaction volume and the long reaction–diffusion pathway will greatly prolong the reaction time because the long diffusion distance from the solution to the surface of electrode resulting the long distance and traveling time of the organic molecules. Thus, a PEC thin-layer reactor has been chosen in this work to study the adsorption and degradation performance of organics.

### The Adsorption and Degradation Characteristics of Different Organics on TNA

**Fig. 4:**
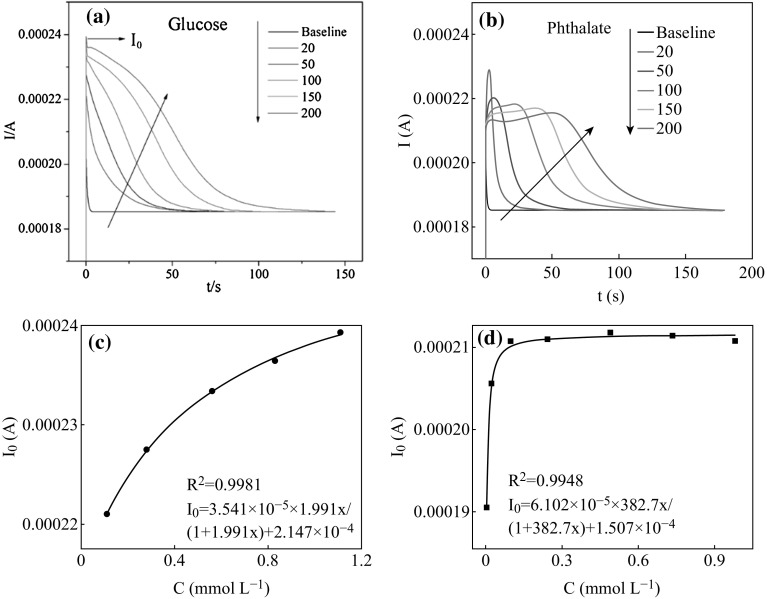
*I*–*t* curves obtained from PEC degradation of **a** glucose and **b** phthalate at different concentrations. The relationship between *I*_0_ value and the initial concentration of **c** glucose and **d** phthalate

Glucose and phthalate were chosen as the target substrates for the PEC degradation experiments. Glucose and phthalate represent the carbohydrate and aromatic acid compounds, respectively. Figure 4a, b shows the *I*–*t* curves, which immediately reflected the characteristics of the organics PEC degradation, obtained from the PEC degradation of glucose and phthalate, respectively. It can be seen that these two types of organics display different degradation properties. Figure 4a shows the *I*–*t* curves obtained from the glucose degradation; all this set of *I*–*t* curves achieves the peak values as soon as the reaction starts, and then quickly descends over time with a relatively simpler attenuation trend. With the increasing of the glucose concentration, the peak areas of *I*–*t* curves extend, the oxidation time prolongs, and the photocurrent response values are significantly improved. Obvious elevation of the photocurrent response values at different concentrations have been recorded since the beginning of the PEC degradation. Meanwhile, a curve shape change appears with the increasing of the glucose concentration. For example, the *I*–*t* curve of glucose at 20 mg L^−1^ shows relatively simple trend that rapidly descends after reaching the original photocurrent peak value which illustrates the diffusion process from the bulk solution to the electrode surface was the major factor that affects the oxidation at low concentrations. When the concentration of glucose is up to 200 mg L^−1^, a convex shape-liked trend appears. This phenomenon may be caused by the quick complement of the high concentration gradient. From Fig. 4b, it can be seen that the *I*–*t* curves of phthalate PEC degradation show obvious different tendency compared with glucose. The photocurrent values of phthalate take a period of time to achieve the maximum values, and then decay to the stable stage over the degradation of phthalate. At the low concentration of 20 mg L^−1^, the *I*–*t* curve takes short time to achieve the peak value and then quickly descends with the simple attenuation trend. At the high concentration, e.g., 200 mg L^−1^, the *I*–*t* curve maintains at a certain level and continues to increase until achieving the peak value, and then descends over time. In comparison, the degradation of glucose takes the shorter time to achieve the stable state than that of phthalate. For example, 100 mg L^−1^ glucose takes approximately 80 s, while phthalate takes 130 s to achieve the stable state at the same condition. It can be indicated that glucose is relatively easily to be degraded, while phthalate is hardly to be degraded.

This difference between *I*–*t* curves obtained from the PEC degradation of glucose and phthalate may induce by the different adsorption abilities. Presuming that the adsorption of organic on the surface of TiO_2_ is the monolayer, and the adsorption process completes in the equilibration time before the PEC degradation begins, then the adsorption agrees with the pseudo Langmuir isotherm equation. According to our previous work [25], the relationship between the instantaneous photocurrent value *I*_0_ (as shown in Fig. 4a) and the molar concentration of organic agreed with the pseudo Langmuir isotherm equation:

6 $$ I_{0} = abx/\left( { 1 { } + bx} \right) \, + \, c, $$

where *a* is the Langmuir current response constant, *b* is the adsorption equilibrium constant of organics on the interface, and *c* is the polarization current (A). Accordingly, the relationship between *I*_0_ and concentrations of glucose and phthalate is shown in Fig. 4c, d, respectively. The *R* ^2^ values of glucose and phthalate are 0.9981 and 0.9948, respectively, indicating the good agreement with Eq. (6). The *b* value is an important parameter, equivalent to the adsorption constant of the electrode, and reveals the quantity of the molecular involved into the reaction. It can be seen that the *b* value for glucose and phthalate is 1.991 and 382.7, respectively. The much higher *b* value of phthalate indicates its stronger adsorption property than glucose, that is the more molecule of phthalate may adsorbed on the surface of TNA than glucose under the same condition. Accordingly, phthalate could quickly supply to the PEC degradation on the surface of the TNA electrode since it could be tightly adsorbed on the electrode surface, which reflecting on the *I*–*t* curve is the continuing increase before achieving the peak value. However, the *I*–*t* curves of glucose show relative simpler descend tendency.

### The Adsorption and Degradation Characteristics of TBA on TNA

TBA is a kind of tertiary alcohol, its hydrogen atom and oxygen atom in –OH are firmly bonded due to the high electron cloud density. Moreover, there is no hydrogen atom on the carbon atom which attached to the –OH, resulting the stable property of TBA that hardly to be oxidized or dehydrogenated. It has been reported that the reaction rate constant between TBA and ^•^OH is 5 × 10^8^ L (mol s)^−1^ and could generate the inert intermediate [27, 28].

**Fig. 5:**
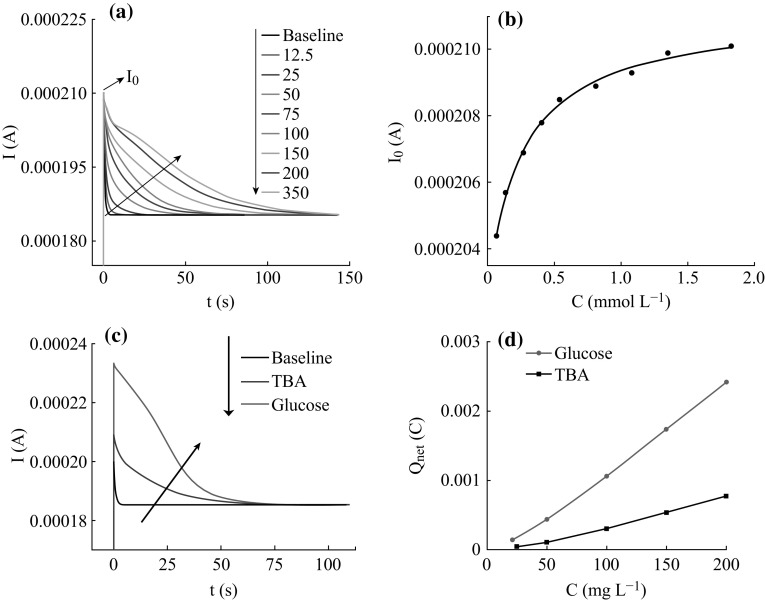
**a** *I*–*t* curves obtained from the PEC degradation of TBA at different concentrations. **b** The relationship between the concentration of TBA and its original photocurrent value. **c** The *I*–*t* curves obtained from the PEC degradation of glucose and TBA both at the concentration of 100 mg L^−1^. **d** The *Q*_net_ obtained from the PEC degradation of glucose and TBA

Figure 5a shows the *I*–*t* curves obtained from the TBA degradation at the concentrations varying from 12.5 to 350 mg L^−1^. All this set of *I*–*t* curves show the similar degradation tendency over time, that is achieving the photocurrent spikes instantaneously in the initial stage (less than 1 s) and then gradually descended over the degradation of TBA. It could be inferred that TBA has been adsorbed onto the surface of TNA electrode before the PEC oxidation started. However, there are still differences among these *I*–*t* curves. That is the photocurrent values increasing, the time takes to achieve the stable state prolonging, and the peak area expanding with the increasing of the concentration.

As mentioned above, TBA is adsorbed onto the surface of the electrode before the PEC oxidation starts, and therefore, the instantaneous photocurrent values could reflect the adsorption and degradation behavior on the surface of the electrode.

Accordingly, the relation between *I*_0_ values and the concentrations (mmol L^−1^) of TBA (shown in Fig. 5b) could be obtained as

7 $$ I_{0} = { 8}. 3 4 4\times 10^{ - 6} \times { 3}. 7 30x \, / \, \left( { 1 { } + { 3}. 7 30x} \right) \,\quad+ { 2}.0 2 8\times 10^{ - 4} R^{ 2} = 0. 9 9 4 7 $$

The correlation coefficient *R* ^2^ = 0.9947 suggests that the relationship between *I*_0_ values, and the concentration of TBA fits well with the Langmuir equation, which actually reflecting the relationship between the instantaneous photocurrent and the adsorbed organic on the surface of the TNA electrode when the quantity of the photogenerated holes is constant. As shown in Fig. 5b, the *I*_0_ values increase with the concentrations of TBA. The *b* value of 3.730 in Eq. (7) indicates the weak adsorption ability of TBA, suggesting the gently translation of TBA molecule from the main solution to the surface of electrode after the degradation of adsorbed molecule, reflecting on the photocurrent is the moderate decay after the quick decline. Table 1 shows the coefficients in Eq. (6) for TBA, glucose, and phthalate. Comparing the *b* values of TBA, glucose, and phthalate, it can be indicated that the adsorption abilities of these three kinds of organics on the surface of TNA are in the order: phthalate (382.7) ≫ TBA (3.730) > glucose (1.991).

**Table 1:** The coefficients of different organics in pseudo Langmuir equation

Value	*R* ^2^	*a*	*b*	*c*
TBA	0.9947	8.344 × 10^−6^	3.730	2.028 × 10^−4^
Glucose	0.9981	3.541 × 10^−5^	1.991	2.147 × 10^−4^
Phthalate	0.9948	6.102 × 10^−5^	382.7	1.507 × 10^−4^

However, the PEC degradation of TBA could not achieve the exhausted mineralization. For illustration, the PEC degradation *I*–*t* curves of TBA and glucose (which could be completely oxidized as mentioned above) at the same concentration of 100 mg L^−1^ are shown in Fig. 5c. It can be seen that the degradations of TBA and glucose take the similar time to achieve the stable state, but both the photocurrent values and the peak area of the *I*–*t* curve obtained from TBA are significantly smaller than that of glucose. It could be inferred that *Q*_net_ of TBA in PEC degradation is much smaller than the corresponding theoretical value. According to Eq. (5), the degradation rate of TBA could be calculated as 28.45%, which suggesting the partly mineralization of TBA. The possible reason is that TBA scavenges the ^•^OH radicals, and the formed inert compound ends the further oxidation.

The *Q*_net_ obtained from the PEC degradation of glucose and TBA at a series concentrations are shown in Fig. 5d. It can be seen that all of the *Q*_net_ obtained from the TBA degradation are much smaller than that of glucose, indicating the partly degradation of TBA at different concentrations in the range of 25–200 mg L^−1^.

### The Inhibition Effect of TBA on the PEC Degradation of Different Organics

**Fig. 6:**
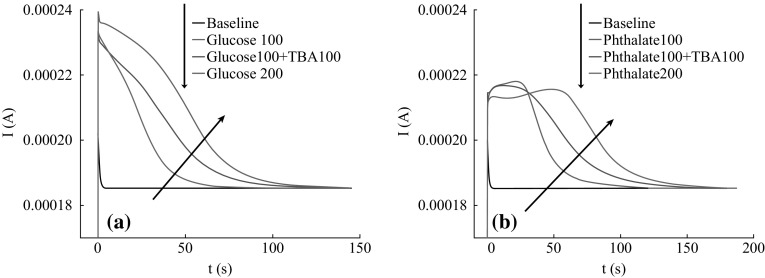
The comparison of *I*–*t* curves obtained from the PEC oxidation of organics in and out of present TBA. **a** Glucose **b** Phthalate

To investigate the effect of TBA on the PEC degradation of organics, 100 mg L^−1^ TBA was added at the concentration ratio of 1:1 into 100 mg L^−1^ different organics to form the organic-TBA mixture with a total concentration of 200 mg L^−1^. For the purpose of comparison, the *I*–*t* curves obtained from the PEC degradation of organics at the concentrations of 100 and 200 mg L^−1^ are also shown in Fig. 6, respectively. Comparing the *I*–*t* curves of 100 mg L^−1^ glucose and phthalate, respectively, containing none and 100 mg L^−1^ TBA, it can be seen that there are no significant differences at the early stage, later the photocurrent values of mixture that containing organic and TBA become higher than that of organics alone, the peak area extends, and the time to achieve the stable state prolongs. However, when comparing the *I*–*t* curves of the organic alone and the mixture both at the concentration of 200 mg L^−1^, there are significant differences. For illustration, the *I*_0_ values for the glucose-TBA mixtures and the glucose alone are 2.326 × 10^−4^ A and 2.394 × 10^−4^ A, respectively. Obviously, the former is much smaller than the latter. This phenomenon may be caused by the occupation of the reaction sites by TBA instead of glucose in the mixture because the adsorption property of TBA is stronger than glucose (see the *b* value shown in Table 1). In addition, the capture of generated ^•^OH by TBA may also affect the instantaneous photocurrent value. However, this difference of the *I*_0_ values between the phthalate and the phthalate-TBA mixtures is not significant due to the obviously strong adsorption capacity of the phthalate avoiding the ^•^OH capture by TBA. Additionally, the photocurrent values and the peak area of glucose-TBA are smaller than that of glucose alone. Although the photocurrent values of 200 mg L^−1^ phthalate are smaller than the mixture, the peak area of 200 mg L^−1^ phthalate is larger than phthalate-TBA mixtures. That means the mixture could not achieve the completely degradation in the presence of TBA. In other words, the TBA inhibits the degradation of the organics.

**Fig. 7:**
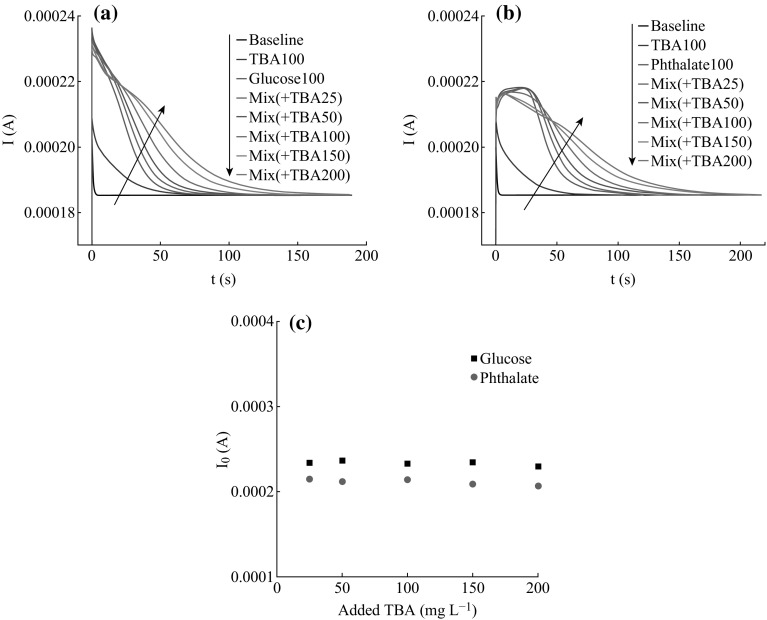
*I*–*t* curves obtained from the PEC degradation of 100 mg L^−1^ TBA and 100 mg L^−1^ **a** glucose **b** phthalate containing TBA of different concentrations, respectively. **c** The relationship between *I*_0_ value and the concentration of TBA that existing in the glucose-TBA and phthalate-TBA mixtures

The effects of different concentrations of TBA on glucose and phthalate of 100 mg L^−1^, respectively, were investigated in this work. The *I*–*t* curves of organics containing a series TBA are shown in Fig. 7a, b. It can be seen that the *I*_0_ values obtained from glucose-TBA mixture do not notably increase, even there are slightly decrease, with the increasing of TBA concentration as soon as the PEC degradation starts, which are very different from the display of glucose alone. In the late stage, the *I*–*t* curve values become to increase, the peak areas extend, and the degradation times prolong with the increasing of TBA concentration. Figure 7b shows the *I*–*t* curves obtained from the phthalate-TBA mixtures, the degradation process could be divided into three stages according to the obvious differences among this set of curves. At the initial state, the curves almost overlay. Later, the reduction of the photocurrent values appears and aggravates with the increasing of TBA concentration. Finally, the photocurrent values increase with the increasing of TBA concentration, the peak area extends, and the time to achieve the stable state prolonged. For concrete description, Fig. 7c shows the *I*_0_ values of both series of the glucose-TBA mixtures and the phthalate-TBA mixtures. It can be seen that the *I*_0_ values do not increase with the concentration of the added TBA which is different from the PEC degradation of each organic alone (see in Fig. 4c, d). This phenomenon may due to the instance photocurrent responses of TBA are not dominant in the mixture; for illustration, the *I*_0_ value of 100 mg L^−1^ TBA is 2.085 × 10^−4^ A, much smaller than that of glucose (2.330 × 10^−4^ A) and phthalate (2.107 × 10^−4^ A) at the same concentration. Even though TBA affects the PEC degradation of both glucose and phthalate on the surface of the TNA, the current values in the period of beginning decrease with the increasing of the TBA concentrations as mentioned above.

**Fig. 8:**
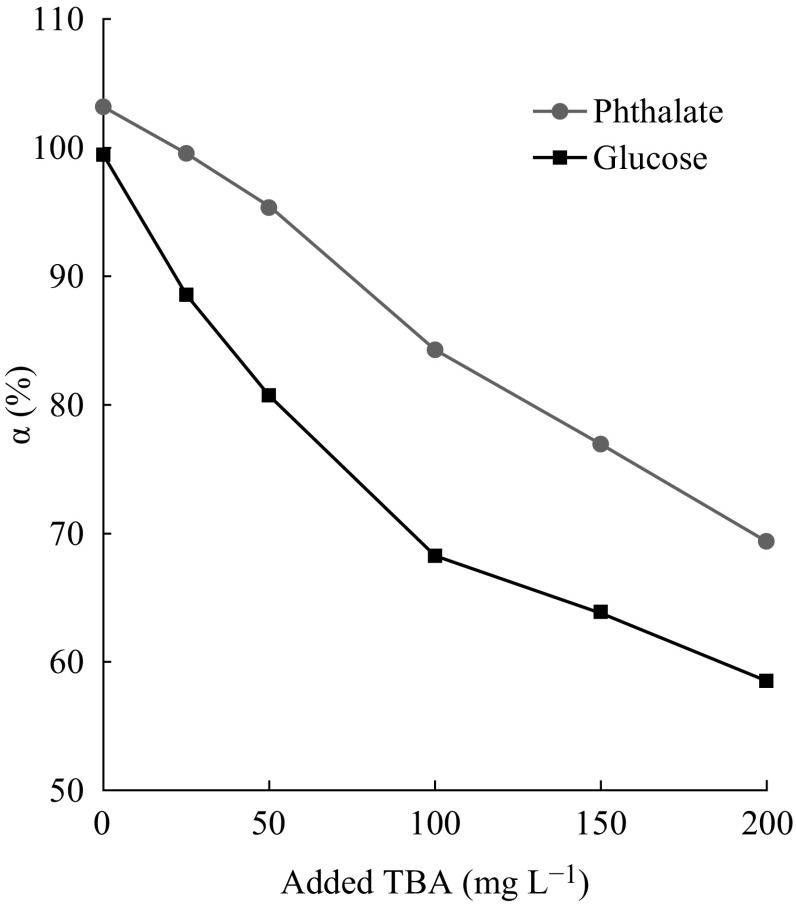
The degradation rate of the mixture *vs* the different concentrations of added TBA

However, the degradation rate (shown in Fig. 8) of glucose-TBA and phthalate-TBA calculated according to Eq. (4) is much smaller than 100 %, even decreased with the increasing of TBA concentrations. It can be indicated that the organics in the solution have not been exhaustedly mineralized in the presence of TBA. In addition, the degradation rates of both sets are decreased with the increasing of TBA concentration. It suggests the inhibition effect on organics enhanced with the increasing of the TBA. Comparing these two sets of data, it can be seen that the degradation rates of glucose-TBA mixture are smaller than that of phthalate-TBA mixture under the same TBA concentration. That means the inhibition effect of TBA is varied with the kind of organic.

### The Inhibition Mechanism of TBA on the Surface of TNA in the PEC Degradation

The adsorbed organics on the surface of the TNA electrode are oxidized by the photogenerated holes in advance as soon as the PEC degradation starts. Considering the adsorption ability varies with the species of organics, there must be competition between two different kinds of organics existing in the solution, resulting inhomogeneous distributions of adsorption on the surface of TNA electrode.

**Fig. 9:**
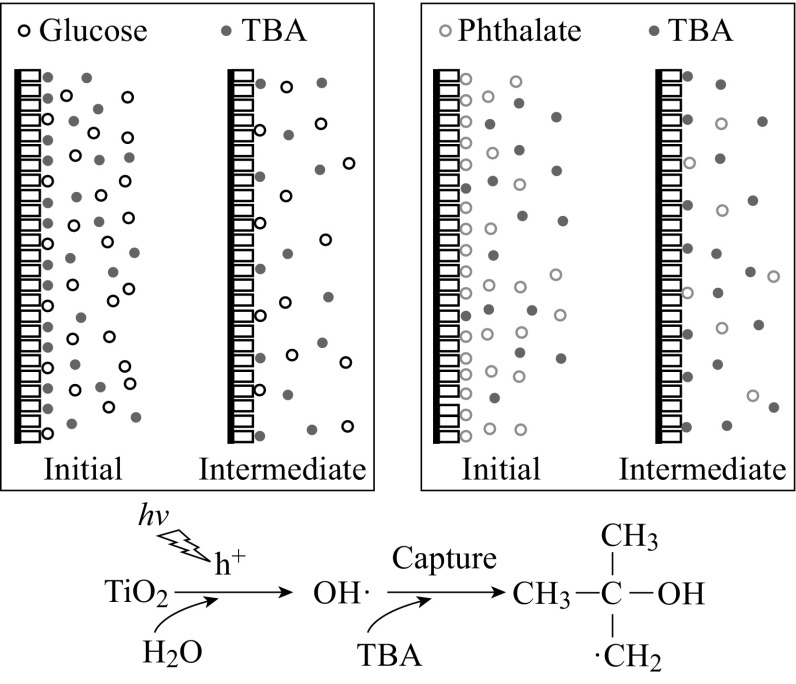
The schematic diagram of the PEC degradation of organic-TBA mixtures on the surface of TNA electrode

Take the glucose-TBA mixture as the example, the quantity of adsorbed TBA on the surface of TiO_2_ is more than that of glucose because the adsorption ability of TBA is stronger than glucose. Thus, the degradation of TBA is slightly superior than glucose at the initial state of PEC degradation process. Then, the molecules of glucose and TBA move to the surface of the electrode and could be degraded over time. In this process, TBA is known as the ^•^OH radical scavenger which could react with the ^•^OH radicals to form the inert intermediate that leading to the termination of the PEC degradation. These ^•^OH radicals will react with TBA that adsorb on the surface of the TNA electrode to form the inert compounds which could be hardly oxidized in the continuing PEC degradation. Another possible inhibition aspect may relate to the polyhydroxy molecular structure of glucose that may generate the ^•^OH radical in the PEC process, then diffuse into the main solution body to react with TBA there (see Fig. 9).

In comparison, the adsorption coefficient of phthalate is significantly higher than that of TBA. Thus, large amount of phthalate, which has the absolute advantage in the distribution, adsorbs on the surface of the electrode within the equilibration time. Therefore, the major oxidation object of photogenerated holes and ^•^OH radicals is phthalate when the PEC degradation begins. As the PEC degradation proceeds, phthalate and TBA in the solution body transfer to the surface of the electrode along with the degradation of the adsorbed organics. In the transition process, the speed of phthalate is faster than TBA because of its strong adsorption property. So the transition and the degradation of TBA are gradually becoming the major reaction as the phthalate concentration decreasing. In other words, the degradation of TBA in the mixture of phthalate-TBA is continue to occur and gradually enhanced. According to the above mentioned, the inhibition effect of TBA is greater on glucose than phthalate which could be inferred from the degradation rate of the mixtures.

More TBA will distribute on the surface of the electrode with its increasing of concentrations, resulting the more notable inhibition effect on the PEC degradation of organics. This phenomenon could be certified by the degradation rates of both the glucose-TBA and phthalate-TBA mixtures shown in Fig. 8.

## Conclusions

The inhibition effect of TBA on the PEC degradation of different organics on the surface of TNA and its mechanism were studied by using a thin-layer reactor. Glucose and phthalate were chosen as the object organic matters. The results showed that both glucose and phthalate, with concentrations ranging from 0 to 200 mg L^−1^, could be exhaustedly mineralized, but a shorter degradation time was taken by glucose at the same concentration. TBA, however, could hardly be completely degraded under the same condition. The adsorption properties of different organics were also studied by the instantaneous photocurrent values, and the adsorption coefficients of TBA, glucose, and phthalate were 3.730, 1.991, and 382.7, respectively. The degradation of both glucose and phthalate could be inhibited evidently by TBA, which was identified as the ^•^OH radical inhibitor. The different inhibition effects of TBA on glucose and phthalate could be attributed to the differences in the adsorption property and the degradation mechanism on the TNA photoanode.
